# Identification of longitudinally dynamic biomarkers in Alzheimer’s disease cerebrospinal fluid by targeted proteomics

**DOI:** 10.1186/1750-1326-9-22

**Published:** 2014-06-06

**Authors:** Kristin R Wildsmith, Stephen P Schauer, Ashley M Smith, David Arnott, Yuda Zhu, Joshua Haznedar, Surinder Kaur, W Rodney Mathews, Lee A Honigberg

**Affiliations:** 1 Department of Phamacodynamic Biomarkers within Development Sciences, Genentech, Inc. (a member of the Roche Group), 1 DNA Way, South San Francisco, CA 94080, USA; 2 Department of Protein Chemistry within Research, Genentech, Inc. (a member of the Roche Group), 1 DNA Way, South San Francisco, CA 94080, USA; 3 Department of Nonclinical Biostatistics within Product Development, Genentech, Inc. (a member of the Roche Group), 1 DNA Way, South San Francisco, CA 94080, USA; 4 Department of PKPD within Development Sciences, Genentech, Inc. (a member of the Roche Group), 1 DNA Way, South San Francisco, CA 94080, USA; 5 Department of Bioanalytical Sciences within Development Sciences, Genentech, Inc. (a member of the Roche Group), 1 DNA Way, South San Francisco, CA 94080, USA

**Keywords:** Alzheimer’s disease, Cerebrospinal fluid, Biomarkers, Mass spectrometry, LC-MS, Targeted-proteomics, Multiple-reaction monitoring

## Abstract

**Background:**

Alzheimer’s disease (AD) is the leading cause of dementia affecting greater than 26 million people worldwide. Although cerebrospinal fluid (CSF) levels of Aβ_42_, tau, and p-tau_181_ are well established as diagnostic biomarkers of AD, there is a need for additional CSF biomarkers of neuronal function that continue to change during disease progression and could be used as pharmacodynamic measures in clinical trials. Multiple proteomic discovery experiments have reported a range of CSF biomarkers that differ between AD and control subjects. These potential biomarkers represent multiple aspects of the disease pathology. The performance of these markers has not been compared with each other, and their performance has not been evaluated longitudinally.

**Results:**

We developed a targeted-proteomic, multiple reaction monitoring (MRM) assay for the absolute quantitation of 39 peptides corresponding to 30 proteins. We evaluated the candidate biomarkers in longitudinal CSF samples collected from aged, cognitively-normal control (n = 10), MCI (n = 5), and AD (n = 45) individuals (age > 60 years). We evaluated each biomarker for diagnostic sensitivity, longitudinal consistency, and compared with CSF Aβ_42_, tau, and p-tau_181_. Four of 28 quantifiable CSF proteins were significantly different between aged, cognitively-normal controls and AD subjects including chitinase-3-like protein 1, reproducing published results. Four CSF markers demonstrated significant longitudinal change in AD: Amyloid precursor protein, Neuronal pentraxin receptor, NrCAM and Chromogranin A. Robust correlations were observed within some subgroups of proteins including the potential disease progression markers.

**Conclusion:**

Using a targeted proteomics approach, we confirmed previous findings for a subset of markers, defined longitudinal performance of our panel of markers, and established a flexible proteomics method for robust multiplexed analyses.

## Background

Alzheimer’s disease (AD) is the most common form of dementia and the 6^th^ leading cause of death in America [1]. The pathological hallmarks of AD are extracellular plaques composed of amyloid beta (Aβ) and neurofibrillary tangles composed of hyperphosphorylated tau (p-tau). Reductions in cerebrospinal fluid (CSF) Aβ_42_ and increases of CSF tau and p-tau_181_ are observed in AD patients in comparison with aged, cognitively-normal individuals, and these changes in CSF correlate with the pathological hallmarks of AD [2,3]. The change in levels of Aβ_42_ and tau begin 1–2 decades prior to onset of symptoms, with the change in Aβ_42_ preceding that of tau [4,5].

CSF Aβ_42_, tau and p-tau_181_ are routinely used in AD research and drug development. However, while the rank order performance (relative quantitation) of these biomarkers has been consistent, the inter-laboratory variability in the absolute value has been high [6-8]. In addition to variable performance, there is significant overlap between levels in controls and AD patients. Further, none of the three established CSF biomarkers have been directly linked to efficacy in a clinical trial. For example, while CSF levels of tau and p-tau_181_ levels did trend toward decline after treatment with bapineuzumab, no correlation with cognitive benefit was observed (reviewed in [9]).

There is a need to develop additional biomarkers that can aid in the diagnosis and treatment of AD. Many proteomic studies have identified potential AD biomarkers (reviewed by [10,11]), generating a substantial list of candidates that need to be evaluated for their utility. However, follow-up studies focusing on individual candidates are limited and usually focused on markers for which existing immunoassays are available. The development time for a new immunoassay can be significant and relies on successful identification of specific and high affinity antibodies. Targeted-proteomics is an emerging tool to accelerate hypothesis driven biomarker assay development by enabling the quantitative assessment of potentially hundreds of biomarkers simultaneously (reviewed in [12-14]).

Candidate AD biomarkers in CSF were selected based upon the consistency of their identification in proteomic discovery experiments [15-23]. These experiments used many different proteomic approaches, including differences in sample preparation (in-gel vs. in-solution digestion), the mass spectrometer (MS) used for analysis (MALD-ToF vs. FT-ICR), and the quantitation technique (label-free vs. iTRAQ). The differences in the proteomic techniques may account for some discrepancies in the trend of change observed for certain markers, but more importantly highlight the need for targeted-follow-up of candidate biomarkers within a single sample set. Independent of the proteomic experiments, additional candidate biomarkers were selected for evaluation based on their known relationship with AD (e.g. apolipoprotein E4) [24], or their emerging promise as disease progression markers (ex. Visinin-like-protein 1) [25,26].

A multiplexed, absolute quantitative LC-MS/MS multiple reaction monitoring (MRM) assay was developed to quantitate candidate biomarker-specific peptides in CSF samples from cognitively-normal, aged control, mildly-cognitively impaired (MCI) and AD patients. We evaluated the diagnostic utility of all the protein-specific peptides in comparison with the published proteomic literature and also compared with the classic AD CSF biomarkers Aβ_42_, tau and p-tau. The longitudinal performance of each biomarker (sampled serially from the same patient) was established, with the majority of peptides demonstrating stability over the course of a year. Four candidates decreased over time in AD patients. Results from this targeted-proteomic assay narrow the list of candidates for more rigorous follow-up and increase the rate of development of novel clinical biomarkers for AD.

## Results

### CSF biomarker MRM panel development

Based on a review of the CSF discovery proteomics and biomarker literature, initially 50 candidate biomarkers were selected for evaluation. Of these, 30 proteins were detectable in pooled cynomolgus monkey and pooled human CSF (from young-cognitively normal control and Alzheimer’s subjects) by a high resolution LTQ-orbitrap MS operating in unbiased, discovery mode (Table 1). Representative peptides for each biomarker candidate were selected based upon the robustness of their detection in CSF. For example, the quantitation of 41 peptides was compared in three AD CSF samples after one or two freeze thaws. The freeze thaw performance was of special importance for this experiment because of different collection protocols used for the AD CSF samples; some of the AD samples underwent one more freeze-thaw cycle than the other samples analyzed in this study. The 39 peptides selected in the final MRM panel include peptides from the 30 detectable proteins as well as peptides from blood contamination markers, a non-endogenous internal standard, and one CSF protein which was undetectable in the discovery experiment. The peptides are listed in Additional file 1: Table S1; they demonstrated no difference in peptide performance between 1 vs. 2 freeze-thaw cycles (Additional file 2: Figure S1, Pearson r = 0.9938). Peptides with intra-assay and inter-assay CVs of less than 20% were analyzed in patient samples (a summary of peptide performance is shown in Additional file 1: Table S1. LODs, LOQs and % CV was determined from the performance of 4 different calibration curves prepared on different days run in duplicate or triplicate). For the majority of proteins only one signature peptide was selected, with a few exceptions for lower abundance proteins or proteins for which specific peptides (i.e. isoform-specific) were previously published or known to have significance to disease (i.e. APOE4). When possible, species conserved peptides were selected (ex. cynomolgus monkey to human). Peptides were quantitated using the AQUA approach [27,28]; briefly, stable-isotope peptides for each candidate-peptide were synthesized and used as internal standards. The ratio of the light (endogenous) to heavy (stable-isotope-labeled) peptide was mapped to an external calibration curve of known ratios of pure light to heavy peptides. In addition, a non-endogenous protein (horse myoglobin) was spiked into CSF at the beginning of the sample preparation to serve as a quality control measure for sample processing [29].

**Table 1:** Selected CSF AD biomarker candidates

**Uniprot ID**	**Identifier**	**Protein**	**Link to AD**	**Change in AD**
P01009	A1AT	α-1-antitrypsin	Neuroinflammation	[16-18,30-33]
P04217	A1BG	α-1-beta-glycoprotein	Unknown	[17,20,34]
P02768	ALBU	Albumin	Aβ polymerization	[17,18]
P05067	A4	Amyloid precursor protein	Aβ peptide precursor	[35]
P51693	APLP1	Amyloid precursor-like protein 1	Beta and gamma secretase substrate	[36]
P02649	APOE	Apolipoprotein E	Apolipoprotein, Aβ clearance	[15,17,18,20,33,37]
Q8TCZ8	APOE4	Apolipoprotein E4	Apolipoprotein, risk factor for AD	[22]
P02749	APOH	Apolipoprotein H (beta-2-glycoprotein 1)	Apolipoprotein	[15,20,21]
P61769	B2MG	Beta-2-microglobulin	Aβ-binding molecule	[15,17,19,20,22,37-41]
P00450	CERU	Ceruloplasmin	Antioxidant	[34,42,43]
P36222	CH3L1	Chitinase-3-like protein 1 (YKL-40)	Neuroinflammation	[15,20,22,23,44]
P10645	CMGA	Chromogranin A	Neurodegeneration	[15,19,20,22,41,45-47]
P10909	CLUS	Clusterin (ApoJ)	Apolipoprotein, Aβ clearance	[15-18,22,33,47]
Q12860	CNTN1	Contactin 1	Neurodegeneration	[16,18]
Q02246	CNTN2	Contactin 2	Neurodegeneration	[16,18]
P01024	CO3	Complement component C3	Neuroinflammation	[18,45,48,49]
P0C0L4	CO4	Complement component C4	Neuroinflammation	[16,22,34,49]
P01034	CYTC	Cystatin C	Aβ-binding molecule	[15,19,20,22,32,38,41,47]
O95502	NPTXR	Neuronal pentraxin receptor	Neurodegeneration	[15,18,50]
Q92823	NRCAM	NrCAM	Neurodegeneration	[22,45,51]
P00747	PLMN	Plasminogen	Aβ clearance	[18]
P04156	PRIO	Prion protein	β Secretase BACE1 inhibitor	[39,40,52]
P41222	PTGDS	Prostaglandin-d2 synthase	Aβ binding protein	[16,20]
P02753	RET4	Retinol binding protein	Unknown	[15,17,18,37,39,40,53,54]
P08294	SODE	Superoxide dismutase	Target of oxidative damage in AD	[15,34]
P05452	TETN	Tetranectin	Neurodegeneration	[15,16]
P02787	TFRE	(sero)transferrin	Oxidative damage in AD	[15,17,34,39,40]
P02766	TTHY	Transthyretin	Aβ-binding molecule	[15,17,18,22,37,39,40,54-57]
P62760	VISL1	Visinin-like protein 1	Neurodegeneration	[25,26,58]
P02774	VTDB	Vitamin D Binding protein	Aβ-binding molecule	[15,18,39,40]

### Diagnostic evaluation

**Figure 1:**
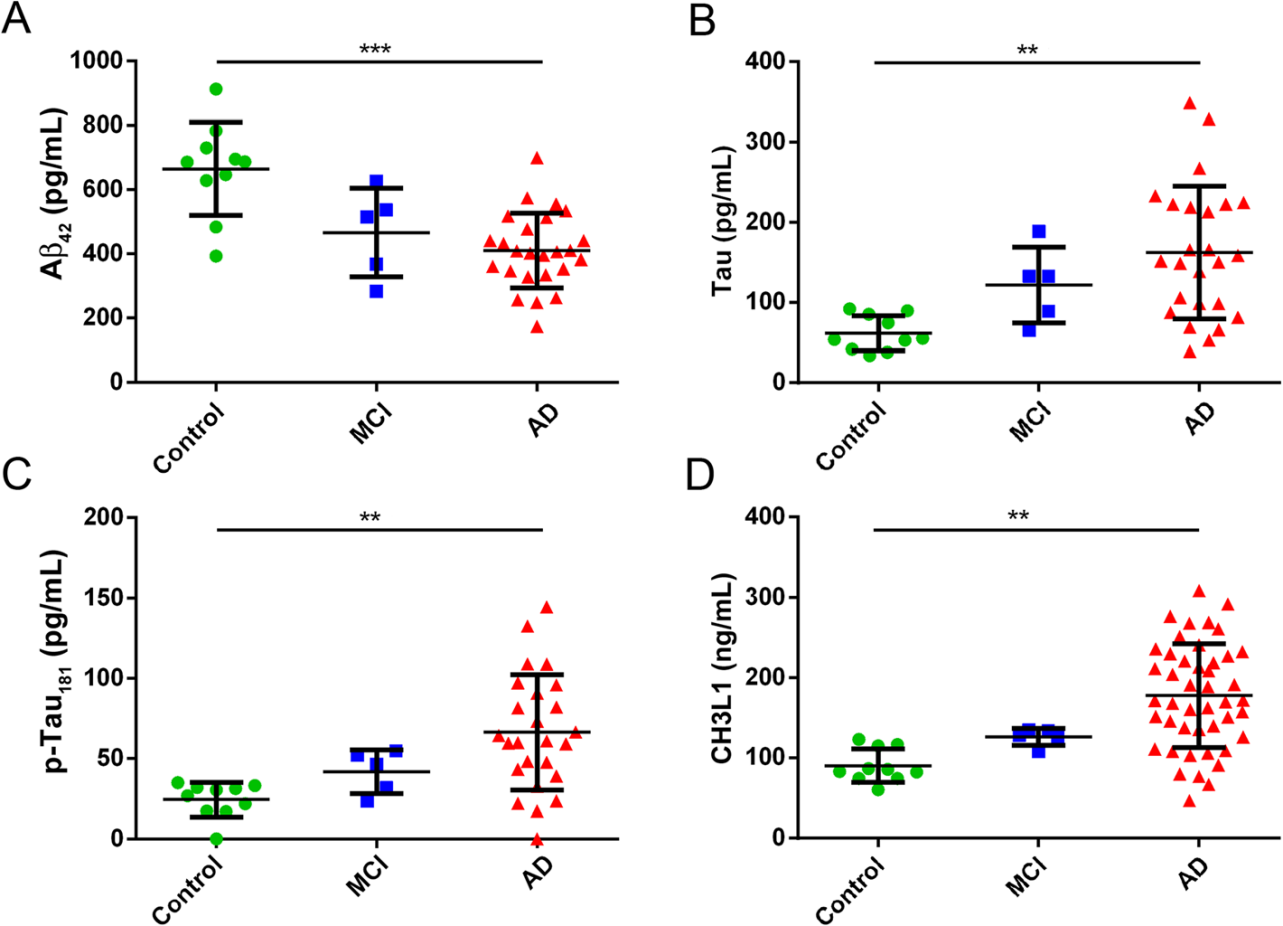
**Difference in Chitinase-3-like protein 1 (CH3L1 aka YKL-40) is comparable with the change observed for Aβ**_**42**_**, tau and p-tau**_**181 **_in AD vs. aged cognitively-normal controls (age > 60y) (linear regression, **p = 0.001-0.01, ***p < 0.001). Control, green-circle, MCI blue-square, AD red-triangle. **A**. CH3L1, **B**. Aβ_42_, **C**. total tau, **D**. p-tau_181_.

Thirty-nine peptides (Additional file 1: Table S1) were quantitated in baseline CSF tryptic-digests from 10 aged (>60 y), cognitively-normal control, 5 MCI, and 45 AD subjects. The CSF sample demographics are summarized in Table 2. Mini-mental state exam (MMSE) cognitive scores were significantly different between control and MCI and control and AD subjects (t-test, p < 0.001), and were consistent with the clinical diagnosis provided by the vendor (Table 2). Similarly, the trends observed for the classic CSF biomarkers Aβ_42_, total tau, p-tau_181_ were consistent with diagnosis (Figure 1A-C). There was a significant difference between the mean age of the groups (control vs. AD p < 0.005, control vs. MCI p < 0.05), so it was important to include as a covariate in our analyses. Four peptides were significantly different between control and AD subjects (linear regression of log values adjusted for age and sex, p < 0.05, corrected by the Benjamini & Hochberg method) (Table 3, Additional file 3: Figure S2). Only Chitinase-3-like protein 1 (CH3L1 aka YKL-40) was at or above the significance level of the common diagnostic biomarkers Aβ_42_, total tau, p-tau_181_ and the MMSE cognitive scores. CH3L1 increased in AD by 1.6-fold (Figure 1D), which is similar to the degree of change observed for Aβ_42_ and tau in these samples (Figure 1A-C). TTHY appears to change in MCI vs. control and 2 biomarkers, PTDGS and APOE_301 reached significance in AD vs. MCI (Table 3, Additional file 3: Figure S2), but due to the low subject number (MCI, n = 5), analysis of a greater number of subjects should be pursued to assess diagnostic potential.

**Table 2:** Demographics

**Characteristics**	**Cognitively normal**	**Mild cognitive impairment**	**Alzheimer’s disease**
n	10	5	45
Sex, M/F	7/3	2/3	30/15
Age, median	68.5	76	77.5
Age, mean (range)	68.8 (64–75)	74 (66–80)	76.9 (61–90)
MMSE score, median	30	24	20
MMSE score, mean (range)	29.4 (25–30)	23.4 (21–26)	19.7 (6–27)

**Table 3:** Summary of biomarkers that differ at baseline between diagnostic groups

	**Group comparison**					
	**AD/Ctl**		**MCI/Ctl**		**AD/MCI**	
**Biomarker**	**p-value**	**Fold difference**	**p-value**	**Fold difference**	**p-value**	**Fold difference**
*MMSE*	*<0.001*	*0.63*				
*Aβ42*	*<0.001*	*0.60*				
CH3L1_290	0.003	1.6				
*Total Tau*	*0.004*	*2.0*				
TTHY_56	0.006	1.2	0.04	1.2		
*p-tau181*	*0.007*	*2.1*				
A4_117	0.031	0.7				
CO3_1172	0.031	1.4				
PTGDS_23					0.034	0.82
APOE_301					0.049	0.73

p values represent linear regression comparison of log values (corrected by the Benjamini & Hochberg method), adjusting for age and sex, and the table includes all measurements with p < 0.05. Fold difference reflects ratio of the average baseline measurements between groups. Differences for MMSE and ELISA measurements of Aβ42, total tau and p-tau are included for reference (*italicized*). (AD n = 45, Ctl n = 10, MCI n = 5, aged >60y).

### Longitudinal performance

**Figure 2:**
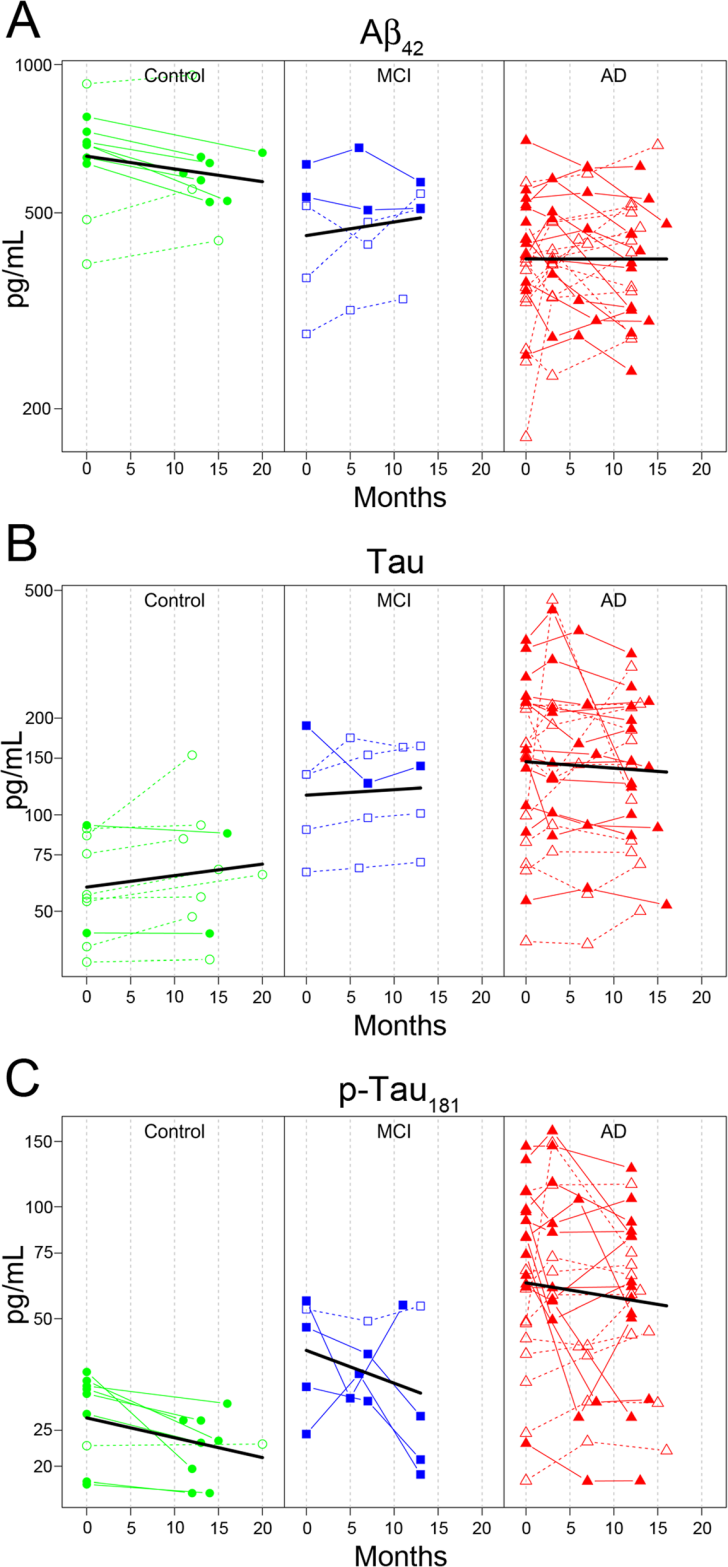
**Classical CSF biomarkers of AD are stable in established disease.** Black-line mean slope. Control, green-circle, MCI blue-square, AD red-triangle. Closed symbols, decliners. **A**. Aβ_42_, **B**. total tau, **C**. p-tau_181_.

**Figure 3:**
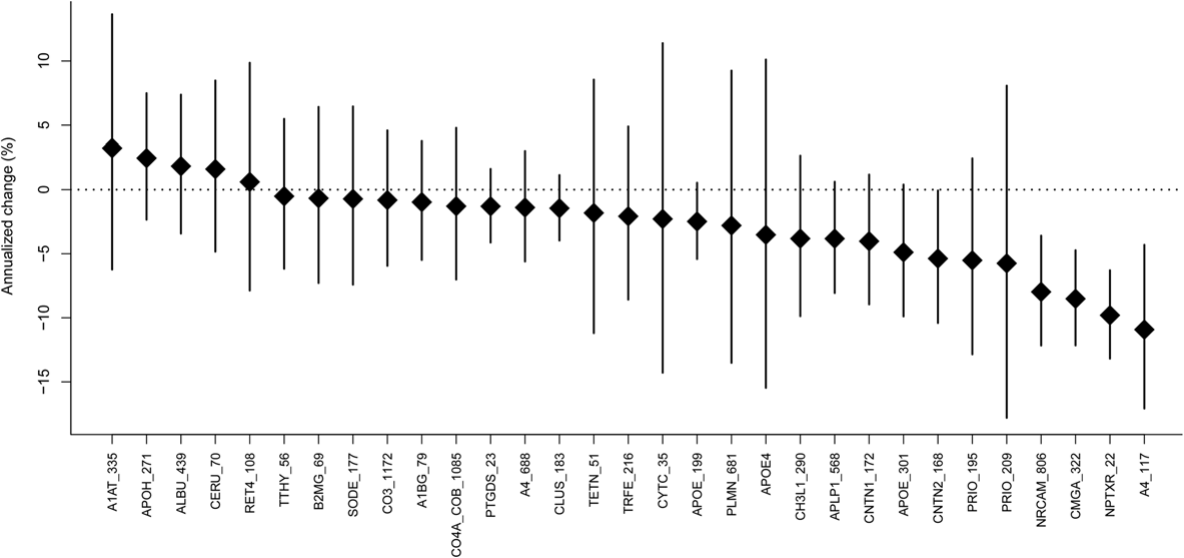
**Estimated annual change of biomarkers in AD patients (n = 45) adjusted for age and sex.** Dot, mean change. Line, 95% confidence interval.

**Figure 4:**
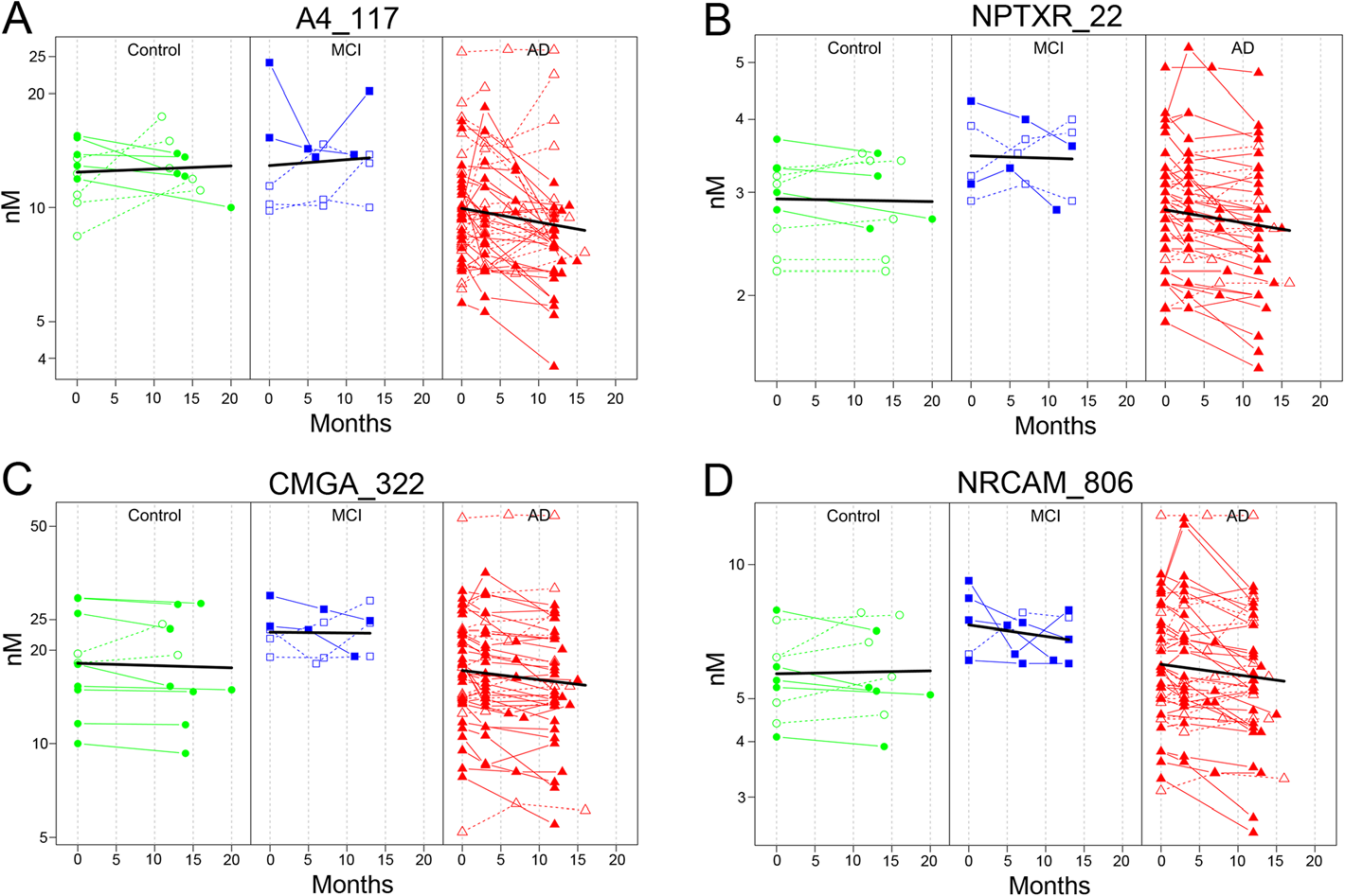
**Potential longitudinal biomarkers in established AD patients.** Black-line mean slope. Control, green-circle, MCI blue-square, AD red-triangle. Closed symbols, decliners. **A**. Amyloid precursor protein peptide (A4_117), **B**. Neuronal pentraxin receptor (NPTXR), **C**. Chromogranin A (CMGA), **D**. NrCAM.

It is estimated that levels of Aβ_42_ and tau change 1–2 decades prior to AD onset [4,5]. However, both markers demonstrate limited to no annual change in established AD patients [59-63]. One of the primary goals of our study was to evaluate the longitudinal stability of the candidate biomarkers. We estimated the annualized rates of change via a linear mixed-effects model using three time points collected repeatedly from the same patients (baseline, 3–8 mo., 11–16 mo.) including age and sex as covariates [64]. As expected, both Aβ_42_ and tau remained stable in the AD subjects’ samples analyzed in this study (Figure 2) (% annual change for Aβ_42_ = −0.1%, 95% CI = −7.3 - 7.7% annual change for tau = −5.4, 95% CI = −16.1-6.6). P-tau trended toward a decrease in AD, but the change from baseline did not reach significance (% annual change for p-tau = −10.8%, 95% CI = −21.4-1.3). The annual rate of yearly change was estimated for all peptides in AD subjects (Figure 3). The majority of peptides were stable over time, however, four peptides demonstrated significant decreases over time in AD as indicated by the 95% confidence interval error bars (~10% per year) (amyloid precursor protein, A4_117; neuronal pentraxin receptor, NPTXR; Chromogranin A, CMGA; and NrCAM) (Figure 3). The individual trajectories and the mean group slope are shown in Figure 4 for the four potential longitudinal biomarkers. There was no significant change from baseline observed in a smaller set of aged control and MCI patients (Figure 4).

### Correlation analysis

**Figure 5:**
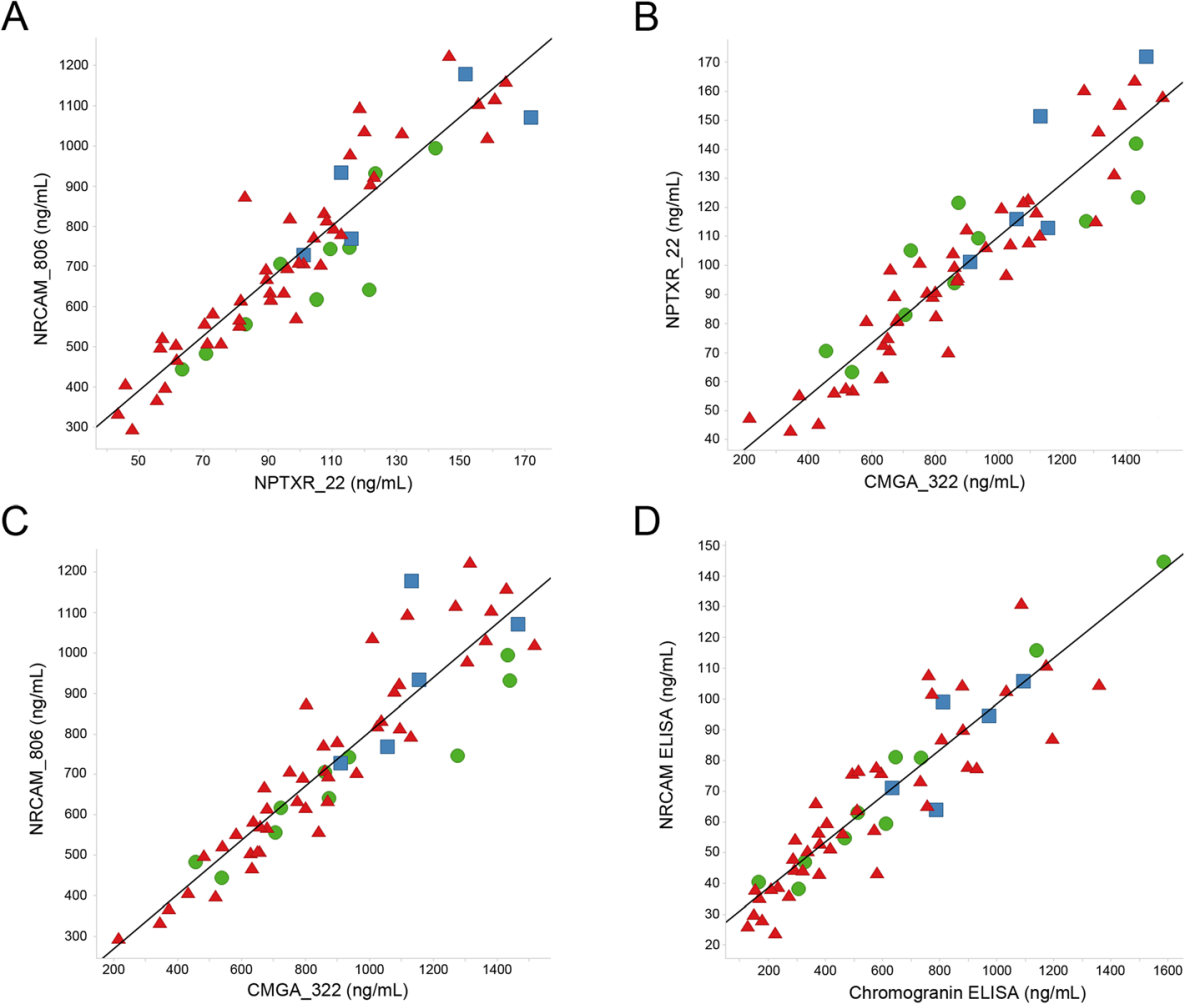
Chromogranin A (CMGA), NrCAM, and Neuronal pentraxin receptor (NPTXR) are highly correlated (A-C), NrCAM and CMGA concentrations are also correlated by ELISA (D) (Baseline for all groups, Control, green-circle, MCI blue-square, AD red-triangle).

The relationship of all peptides with each other as well as with Aβ_42_, tau and p-tau_181_ was compared. Spearman rank correlations were assessed in all samples, and the most significant correlations (|r > 0.8|) are shown in Table 4. Chromogranin A (CMGA), NrCAM and Neuronal pentraxin receptor (NPTXR) were the most highly correlated biomarkers (Figure 5) with correlation coefficients greater than 0.9 for all subjects independent of group, sex, age or time point. When measured by ELISA, CMGA and NrCAM were also highly correlated, and the rank order was consistent with the MRM result (Figure 5D, R = 0.93); thus confirming the observed correlation by an independent method. In addition, within AD patients (baseline) these three tightly correlated peptides did not correlate with levels of CSF total protein (n = 32) or Aβ_42_ (n = 21) (Additional file 1: Table S2), however they did correlate with tau (n = 21)(CMGA R = 0.69, NPTXR R = 0.71, NrCAM R = 0.74) (Additional file 1: Table S2).

**Table 4:** Most significant correlations for all time points and all groups (Spearman) (R > 0.8)

**Peptide X vs. Peptide Y**	**R (Spearman rank)**
CMGA_322 vs. NRCAM_806	0.93
CMGA_322 vs. NPTXR_22	0.93
NPTXR_22 vs. NRCAM_806	0.92
APOE_301 vs. B2MG_69	0.88
CYTC_36 vs. TETN_51	0.88
B2MG_69 vs. TETN_51	0.88
A4_688 vs. NRCAM_806	0.87
B2MG_69 vs. PRIO_195	0.87
CLUS_183 vs. PTDGS_23	0.87
A4_688 vs. CMGA_322	0.87
A4_688 vs. NPTXR_22	0.85
PRIO_195 vs. TETN_51	0.83
APOE_301 vs. PRIO_195	0.83
CERU_70 vs. CO3_1172	0.82
CERU_70 vs. PLMN_681	0.81
APOE_199 vs. APOE_301	0.80

## Discussion

Using a multiplexed, targeted-proteomics approach we evaluated candidate biomarkers for AD that were previously reported in discovery proteomic experiments to change with disease. At least one surrogate peptide was selected for each protein, and 4 specific transitions were selected for each peptide. Stable-isotope labeled peptides (heavy) were used as internal standards for every endogenous peptide (light), and the ratio of the peptide pair (light to heavy) was used for absolute quantitation. Using CH3L1 as an example (Figure 1D), absolute quantitation by this approach yields results similar to that of traditional immunoassays [22,23,44,65].

One of the major benefits of our approach, especially when working in a less-complex matrix like CSF, is that sample preparation does not require sample enrichment approaches. For example, sample enrichment using antibodies, even in the case of depleting the most abundant proteins, such as albumin, can add bias due to co-depletion of proteins of interest [66]. However, enrichment strategies are usually needed when additional sensitivity is required. In this assay, the concentration range in CSF for the 28 proteins detected was 28 ng/mL to 13.8 μg/mL, which is consistent with the expected dynamic range of current instruments. Visinin like protein 1 (VILIP-1) was below the level of detection for all three peptides that were monitored; targeted-enrichment strategies or development of a sensitive immunoassay will be required to evaluate this candidate biomarker.

While not a primary focus of our study due to our small group sizes, we did evaluate the diagnostic performance of all the candidate biomarkers in comparison with the classic AD CSF biomarkers. The directionality and fold-difference observed between diagnostic groups was as expected for Aβ_42_, tau and p-tau in our sample set (Figure 1, Table 3); this result gave us confidence that these samples are representative of their respective diagnostic groups and that they could be used to evaluate the performance of the candidate biomarkers. After correction for multiple comparisons testing, a number of peptides reached significance in differentiating the diagnostic groups (Table 3). However, due to the small group size, replication with more individuals needs to be performed, especially for the MCI group comparisons. The observed modest differences between groups (0.6-2 fold-differences) are comparable with what others have reported; proteomic studies of larger sample sets have demonstrated that the majority of candidates change by less than 2-fold in AD [16,22]. Extending from sporadic AD to familial AD, Ringman et al. also reported that complement component C3 (CO3) increased in disease by 1.3 [34], in comparison with our observed changes of 1.4. Also consistent with previous reports, the traditionally used biomarkers Aβ_42_, tau and p-tau_181_ were the most significant and specific markers differentiating AD patients from control. The other significant biomarkers detected lack specificity; they have also been reported to change in other neurodegenerative diseases including Parkinson’s disease and multiple sclerosis [15,16,67]. The biomarkers evaluated in this study may have greater utility for disease-staging rather than diagnostics [22] or for monitoring therapeutic response, especially if used in combination; if multiple molecules from the same pathway change in response to treatment, it would give one higher confidence of a therapeutic effect.

It is important to characterize the longitudinal stability of a candidate biomarker prior to use in the clinic. Multiple studies have demonstrated that CSF Aβ_42_ levels stabilize and plateau in established AD [59-63]. Similarly CSF tau and p-tau appear to demonstrate longitudinal stability [68], though some studies have noted a slight continued longitudinal increase in tau [60,63]. In our longitudinal assessment of the classic AD biomarkers, Aβ_42_ and tau levels were stable over the course of a year in our AD patients (Figure 2), consistent with previous reports. Similar to Aβ_42_ and tau, the majority of peptides were stable over time (11–16 months) in AD patients (Figure 3). There were four peptides (A4_117, CMGA_322, NPTXR_22, and NRCAM_806) that declined significantly by approximately 10% per year in the AD patients but not in aged control or MCI patients (Figure 4). P-tau trended toward decline over time in 14 of 24 patients (Figure 2). A decline in p-tau (2 pg/mL/year) has been observed previously in sporadic AD patients [69], and more recently in autosomal-dominant AD patients [70]. Fagan et al. also showed that levels of VILIP-1 decreased after the onset of disease [70], suggesting that at later stages of disease a decline in CSF biomarkers, like VILIP-1, are reflective of neuronal loss. The four markers identified in this study are also neuronal markers, and are potential progression markers in Alzheimer’s patients that will be further evaluated in additional longitudinal sample sets.

**Figure 6:**
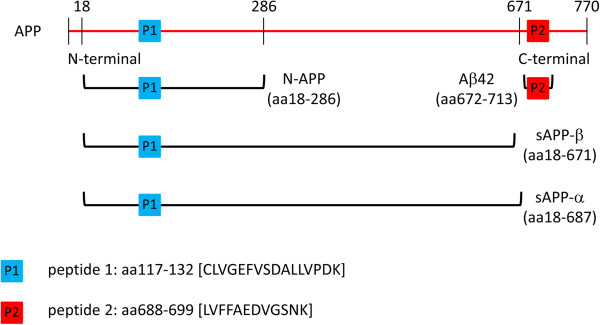
Peptides from different regions of Amyloid Precursor Protein (APP, A4) reflect different processing products.

Interestingly, the potential progression markers CMGA, NrCAM and NPTXR were also highly correlated (Figure 5). The fourth candidate progression marker amyloid precursor protein (APP) peptide A4_117 was also correlated with the three other peptides, however, the Spearman rank correlation coefficient was below our |R > 0.8| cut-off (A4_117 correlations: NrCAM R = 0.65, CMGA R = 0.62, NPTXR R = 0.6). Another APP peptide quantitated in this study, A4_688, did not demonstrate significant longitudinal change. The lack of strong correlation between the two APP peptides, A4_117 and A4_688 (Spearman rank R = 0.42), may be a consequence of differential CSF kinetics of APP cleavage fragments (Figure 6). Based on the full-length sequence of APP A4770, A4_117 corresponds to amino acids (a.a.) 117–132, which is present in soluble APP (sAPP) α (a.a.18-687) and sAPPβ (a.a.18-671) as well as N-APP (a.a.18-286). In contrast, A4_688 corresponds to a.a. 688–699 from full-length APP as well as a.a. 17–28 within Aβ [71]. The differential behavior of these peptides demonstrates the importance of distinguishing peptide-level quantitation from total protein quantitation, and caution against algorithms that automatically combine peptide quantitation into a single protein quantitation.

The correlations were remarkably high for some of these biomarkers and possible analytical factors were evaluated. The majority of potential analytical artifacts were ruled out; for example, peptide properties including retention time and *m/z* were different in each case. To support the hypothesis that the correlations are not an artifact of the detection method, NrCAM and CMGA levels were analyzed using ELISAs. A similar rank order was observed in the MS assay and ELISA (Figure 5D). Interestingly, the absolute levels measured by the NrCAM ELISA were significantly lower than that measured by the MS assay. The difference in absolute value between the NrCAM ELISA and MS result may be due to the fact that NrCAM exists as multiple isoforms in CSF. The antibody used in the ELISA may not detect all the isoforms; however, the peptide used for the MS assay is present in all 5 NrCAM isoforms, which would account for the higher value observed by MS. Although the absolute values were different from the two methods used, the fact that the rank orders are maintained support the hypothesis that the high correlation for some biomarkers reflects a common biological process. CMGA, NPTXR, NrCAM, and A4_117 and A4_688 are all produced in neurons, and changes in these peptides may be reflective of neurodegeneration. NrCAM is an adhesion molecule present on synapses [45,72]. CMGA is a prohormone that is packaged in dense-core synaptic vesicles [46,73,74]. NPTXR and APP are both integral-membrane proteins. NPTXR has been implicated in synaptic plasticity [75,76], and APP is known to be critical to synapse formation and function [77]. Given the diverse biological roles of these proteins, the observed correlations suggest that coordinated changes in CSF may reflect pathologies beyond Aβ and tau (e.g. synaptic function or secretion mechanisms). Interestingly, CMGA, NPTXR, and NrCAM are also positively correlated with tau within our AD samples (R = 0.69, 0.71, 0.74, respectively, Additional file 1: Table S2), although not as strongly as they are correlated with each other. VILIP-1, a potential marker of neurodegeneration, is also positively correlated with tau [26]. Future studies are needed to increase our understanding of the underlying cause of these correlations, the spectrum of other CSF proteins that are correlated with this initial cluster, and the longitudinal performance and utility of the correlations.

## Conclusions

It is now clear that Alzheimer’s disease pathology begins years before the onset of clinical symptoms, and therapeutic trials are beginning to focus on treating patients prior to the onset of dementia. Thus, the need for biomarkers that can increase the accuracy of early diagnosis and reflect the success of treatment is critical for AD researchers, clinicians, and patients. Ideally, biomarkers will be discovered that reflect multiple processes such as neuroinflammation, neuronal stress and neuronal dysfunction, which will enable intervention prior to significant neuronal loss. In addition, biomarkers are needed to monitor progression of disease in established patients. To accelerate the assessment of candidate biomarkers, we developed an absolute quantitative assay for 30 candidate protein biomarkers (Table 1). Biomarkers that were significantly different between diagnostic groups at baseline (fold differences ranging from 0.6-1.6) (Table 3), reproduced published discovery proteomic results. Four of the markers emerged as potential progression markers in AD (Figure 4). Multiplexing enabled the exploration of relationships between CSF biomarkers; we discovered highly correlated proteins that may serve as markers of neurodegeneration in established AD patients (Figure 5). Multiplexed, targeted-proteomics proved to be a robust approach with relatively rapid assay development time enabling the assessment and prioritization of biomarker discovery candidates. This approach promises to fill a much needed gap in clinical biomarker development.

## Methods

### Source of CSF

CSF was purchased from Bioreclamation, LLC (Hicksville, NY) (pooled cynomolgus monkey and pooled human CSF used in discovery experiments), Folio Biosciences (Columbus, OH) (individual longitudinal AD only) and PrecisionMed, Inc. (San Diego, CA) (individual longitudinal Control, MCI and AD). Details on sample collection were provided by the vendors. All donors provided informed consent for use of these studies with institutional review board approval for human collection protocols. CSF was collected in the morning under fasting condition. Lumbar punctures were performed at L3-L4 or L4-5 using a sprotte needle (5 mL), and the CSF was centrifuged and then immediately aliquoted in polypropylene tubes and snap frozen at −80°C. Bloody taps or visually “pink” CSF were excluded. AD or MCI subjects had MMSE between 14 and 28, were greater than 60 years old, Hachinski score ≤4, diagnosed with dementia or MCI established by a clinical examination and documented by MMSE and other neuropsychological tests. Aged cognitively-normal individuals were 64–75 years old, healthy, exhibited normal memory function documented by results within the normal range for CDR word recognition test, and had an MMSE score of greater than 28. The diseased population samples from PrecisionMed were thawed at the vendor for aliquoting, and thus underwent one more freeze-thaw cycle than the other samples analyzed in this study.

### Total protein measurements

Concentrations of CSF total protein were provided by Folio Biosciences as part of the clinical data for each AD subject.

### Immunoassay measurements of CSF Aβ_1–42_, Tau, and p-Tau_181_

INNO-BIA AlzBio3 Kit (Innogenetics®, RUO) was used according to the manufacturer’s protocol. Briefly, beads conjugated to monoclonal antibodies against Aβ_1–42_ (4D7A3), Tau (AT120), or p-Tau_181_ (AT270) were mixed with biotinylated monoclonal antibodies against the same targets (3D6 for Aβ1-42, HT7 for Tau and p-Tau_181_). 75 μL of undiluted CSF was incubated overnight at room temperature with mild agitation. The beads were then washed, and a solution of streptavidin-PE was added to the beads for 1 h to enable detection of bound analytes. After an additional wash, a read solution was added to the beads and they were then analyzed with the Bioplex 200 system (Biorad, Hercules, CA). Only a subset of the AD samples (25 of 45), representative of both vendors, were assayed due to a limited availability of CSF for some subjects.

### CSF tryptic digestion and peptide quantitation

400 μL of CSF was spiked with 100 ng of equine myoglobin (Sigma, St. Louis, MO) and concentrated to 30 μL in a 3 kDa Amicon centrifugal concentrator (Millipore, Billerica, MA). Samples were denatured in 40% trifluoroethanol (TFE) (Sigma) prepared in 100 mM triethylammonium bicarbonate (TEABC) (Sigma) (1 h 37°C). Samples were reduced with 5 mM dithiothreitol (DTT) (30 min RT), alkylated with 20 mM iodoacetamide (IAM) (30 min, RT in the dark), and quenched with an additional 5 mM DTT (15 min, RT). Samples were diluted to 10% TFE with 100 mM TEABC, and then digested with trypsin (1:25, 18 h, 37°C). The digestion was stopped by the addition of formic acid. The final volume of all digests was measured. An aliquot of the total digest (48 uL) was spiked with a mixture of stable-isotope-labeled AQUA peptides (2 uL) (Cell Signaling Technologies, Danvers, MA) prior to LC-MS/MS analysis. Protein-AQUA™ peptides were synthesized by Cell Signaling Technologies and purified by reversed-phase HPLC, and analyzed by MALDI-TOF MS and nanospray tandem MS. An accurate peptide concentration was measured by amino acid analysis, and provide by the vendor. Heavy AQUA peptides to be spiked into samples were diluted and pooled before use to concentrations that were within 10-fold of endogenous protein levels and ranged from 2–100 fmol/uL depending on analyte. In addition, a pool of heavy (stable-isotope labeled) and light (unlabeled) AQUA peptides was prepared and diluted serially to generate calibration curves. The heavy peptide mixture was prepared first. The light peptide was diluted serially using the heavy peptide mixture to hold the concentration of the heavy peptide constant (at the same concentration used for the internal standard spike). Heavy and light peptides used for calibration curves were prepared and qualified in an artificial CSF matrix (bovine serum albumin digest) to select 4 transitions for MRM, establish limits of detection (LOD) and limits of quantitation (LOQ). The observed fmol on-column (2 uL of digest) was converted to nM using a volume correction factor to express the result in relation to the original sample volume. The ranges in human CSF samples and biologic functional roles are summarized in Additional file 1: Table S1 for peptides that demonstrated consistent performance (<20% CV). Values represent the median of 4 different calibration curves prepared on different days run in duplicate or triplicate.

### Discovery LC-MS/MS analysis

Digested proteins from pooled CSF (cynomolgus monkey, human young normal and Alzheimer’s disease subjects) were analyzed by capillary reverse phase liquid chromatography-electrospray ionization tandem mass spectrometry. Aliquots (5 μL of 25 total) were loaded onto the trapping column (BEH C18; 180 μM i.d. × 20 mm with 5 μm particles) of a nanoAcquity (Waters, Milford, MA) ultra high pressure liquid chromatography system and eluted through a resolving column (BEH C18; 100 μm i.d. × 100 mm, 1.7 μm particles) at a flow rate of 1 μL per minute using a linear gradient from 2 to 30% solvent B over 35 minutes followed by a ramp to 50% B in 3 minutes and a step and hold at 90% B for 5 minutes, returning to 2% B for a 7 minute re-equilibration. Solvent A was 0.1% formic acid in water and solvent B was 0.1% formic acid in acetonitrile. Eluted peptides were directed to the electrospray source (CaptiveSpray; Michrom Bioresources, Inc., Auburn, CA) of an LTQ-orbitrap mass spectrometer (Thermo Scientific, Waltham, MA) operated in a “top-8” data-dependent mode whereby high resolution scans of peptide masses in the orbitrap analyzer were followed by ion trap collision-induced dissociation of the 8 most abundant multiply charged ions. Tandem mass spectra were searched against the “UniProt” database of human proteins using the Mascot program (Matrix Science Ltd.) using a “semi-tryptic” enzyme specificity and 25 ppm precursor ion tolerance, with cysteine carbamidomethylation as a static modification and allowing for oxidized methionine as a variable modification. Database hits were filtered to a false discovery rate of less than one percent using a “target-decoy” linear discriminant procedure.

### Targeted LC-MRM analysis

Samples (2 μL) were loaded onto a nanoAcquity UPLC (Waters, Milford, MA), desalted on a Symmetry® C18 trap column (180 μm × 20 mm, 5 μm) (Waters) and separated on a BEH130 C18 (100 μm × 100 mm, 1.7 μm) (Waters) at a flow rate of 1 μL/min over a 60 min. gradient (2%acetonitrile (ACN) 0.1% formic acid (FA) to 30% ACN, 0.1% FA over 40 min; 30-98% ACN, 0.1% FA over 10 min; 98% ACN, 0.1% FA for 5 min; reequilibrate 2% ACN, 0.1% FA). Peptides were detected by a QTRAP® 5500 (AB SCIEX, Framingham, MA) equipped with an Advance Captivespray™ source (Michrom Bioresources, Inc. Auburn, CA). Scheduled MRM methods were prepared using Skyline v1.3 [78] and imported into Analyst 1.5.2 (ABSCIEX). The QTRAP® 5500 was operated in positive ion mode using scheduled MRM. Result files were processed and quantitated using Multiquant™ v2.1 with Scheduled MRM™ algorithm (AB SCIEX), and the results exported for further statistical analysis. Calibration curves were generated using a mixture of light and heavy AQUA peptides spiked into artificial CSF (10 μg/mL bovine albumin digest prepared in the same manner as CSF samples).

### NrCAM and Chromogranin A enzyme-linked immunosorbent assays (ELISAs)

CSF samples were analyzed using commercially available ELISAs for NrCAM (R&D Systems, Inc., Minneapolis, MN) and Chromogranin A (ALPCO Diagnostics, Salem, NH). The assays were performed according to the manufacturers’ instructions. CSF samples were assayed in duplicate and had undergone one previous freeze-thaw cycle after receipt from vendor. For the NrCAM ELISA, CSF samples were diluted 1/64 in Reagent Diluent (Catalog # DY995 R&D Systems) and 100 μL of the diluted samples and kit standards were added per well. Absorbance was measured at 450 nm with wavelength correction at 570 nm on a VersaMax plate reader (Molecular Devices, Sunnyvale, CA). For the Chromogranin A ELISA, CSF samples were diluted 1/4 in CgA Assay Buffer (ALPCO Diagnostics) and 25 μL of the diluted samples and kit standards and controls were added per well. Absorbance was measured at 450 nm with wavelength correction at 595 nm on a VersaMax plate reader.

### Statistical analysis

Results were imported into TIBCO® Spotfire® 4.0.2 (TIBCO® Software Inc, Somerville, MA) and R statistical computing and graphics software. Analyses included data QC for peptide performance (coefficient of variance), QC of sample preparation (equine myoglobin), group comparisons (linear regression, (p-values corrected by the Benjamini & Hochberg method)), longitudinal analysis, and correlation analysis (Pearson and Spearman). Measured “values” were transformed using a log base 10 transformation. Annualized rates of change of “values” were estimated via a linear mixed-effects model [64] implemented with the ‘nlme’ package in R [79] using “patients” as random effect. In both the baseline group comparison model and the longitudinal analysis for annualized rate of change, we adjusted for age as a continuous covariate, sex, and interactions between age and sex.

## Abbreviations

AD: Alzheimer’s disease; CSF: Cerebrospinal fluid; MCI: Mildly-cognitively impaired; MRM: Multiple-reaction monitoring; Aβ: Amyloid beta; p-tau: Phosphorylated tau; LC: Liquid chromatography; MS: Mass spectrometry; ApoE: Apolipoprotein E; MMSE: Mini-mental state exam; CH3L1: Chitinase-3-like protein 1; B2MG: Beta-2-microglobulin; APP: Amyloid precursor protein (A4); NPTXR: Neuronal pentraxin receptor; CMGA: Chromogranin A; CERU: Ceruloplasmin; CO3: Complement component C3.

## Competing interests

All authors are employees of Genentech, Inc. a member of the Roche group, and receive a fixed salary.

## Authors’ contributions

KRW, WRM, LH conceived and designed the experiments. DA performed discovery proteomics LC/MS experiments. KRW developed the LC/MS MRM assay and processed and analyzed CSF samples. SPS and AMS analyzed CSF samples using different ELISAs. JH assisted with data analysis. YZ performed the statistical analysis of all data. KRW, WRM, SK, and LH wrote the paper. All authors edited and revised the manuscript, and gave final approval of the version to be published.

## Supplementary Material

Additional file 1: Table S1 Selected peptides for CSF AD biomarker candidates, peptide performance and biological protein function. **Table S2.** CSF total protein and Aβ_42_ do not correlate with chromogranin (CMGA), neuronal pentraxin receptor (NPTXR) or NrCAM, but tau and p-tau_181_ do (Spearman rank correlations in AD patients at baseline). Click here for file

Additional file 2: Figure S1 The majority of peptides are stable after one or two freeze-thaw cycles. Log of the mean ratio (light to heavy peptide pair) observed for 42 peptides between 1 or two freeze thaw cycles in CSF from three AD patients (shape and color by patient). Click here for file

Additional file 3: Figure S2 Comparison of levels of detectable peptide biomarkers with inter-assay CVs of <20% in aged (>60y) cognitively-normal controls (n = 10), MCI (n = 5), and AD (n = 45) individuals. Differences between control and AD that reached significance are indicated with an asterisk (*p = 0.01-0.05, **p = 0.001-0.01, ***p < 0.001, linear regression comparison of log values corrected by the Benjamini & Hochberg method) (Control, green-circle, MCI blue-square, AD red-triangle). Click here for file
