# Supplementary material for: Identification of longitudinally dynamic biomarkers in Alzheimer’s disease cerebrospinal fluid by targeted proteomics
Source: Mol Neurodegener. 2014 Jun 6;9:22. doi: 10.1186/1750-1326-9-22 (PMC4061120; doi:10.1186/1750-1326-9-22)
Supplement: Additional file 1: Table S1 — Selected peptides for CSF AD biomarker candidates, peptide performance and biological protein function. Table S2. CSF total protein and Aβ42 do not correlate with chromogranin (CMGA), neuronal pentraxin receptor (NPTXR) or NrCAM, but tau and p-tau181 do (Spearman rank correlations in AD patients at baseline). [file 1750-1326-9-22-S1.docx]

**Supplemental Table 1.** Selected peptides for CSF AD biomarker candidates, peptide performance and biological protein function.

| **Uniprot ID** | **Protein** | **Peptide Identifier** | **Selected Peptide** | **LOD (nM)** | **LOQ (nM)** | **Range aged normal CSF (nM)**  **(n=10)** | **Range AD CSF (nM)**  **(n=45)** | **Range aged normal CSF (ng/mL)**  **(n=10)** | **Range AD CSF (ng/mL)**  **(n=45)** | **Protein Function** |
| --- | --- | --- | --- | --- | --- | --- | --- | --- | --- | --- |
| P01009 | a-1-antitrypsin | A1AT_335 | VFSNGADLSGVTEEAPLK | 0.1 | 0.5 | 37-117 | 28-263 | 1232-6268 | 1508-14161 | Protease inhibitor |
| P04217 | a-1-beta-glycoprotein | A1BG_79 | HQFLLTGDTQGR | 0.1 | 1 | 6-17 | 5-27 | 138-1053 | 302-1709 | Unknown |
| P05067 | Amyloid precursor protein | A4_117 | *C*LVGEFVSDALLVPDK | 0.2 | 2 | 7-14 | 4.5-25 | 479-1446 | 455-2474 | Neural plasticity, iron export |
| P05067 | Amyloid precursor protein | A4_688 | LVFFAEDVGSNK | 0.02 | 0.2 | 0.7-1.5 | 0.5-2 | 63-152 | 45-201 | Neural plasticity, iron export |
| P02768 | Albumin | ALBU_439 | VPQVSTPTLVEVSR | 5 | 5 | 54-120 | 47-168 | 3304-9145 | 3557-12856 | Carrier protein |
| P51693 | Amyloid precursor-like protein 1 | APLP1_568 | DELAPAGTGVSR | 0.02 | 5 | 3-5 | 3-6 | 207-421 | 234-504 | Glucose & insulin homeostasis, neurite outgrowth |
| P02649 | Apolipoprotein E | APOE_199 | LGPLVEQGR | 0.2 | 2 | 48-67 | 34-86 | 1609-2799 | 1398-3546 | Lipid transport, neurite outgrowth |
| P02649 | Apolipoprotein E | APOE_301 | VQAAVGTSAAPVPSDNH | 0.1 | 1 | 17-31 | 11-39 | 521-1274 | 443-1603 | Lipid transport, neurite outgrowth |
| Q8TCZ8 | Apolipoprotein E4 | APOE4 | LGADMEDVR | 0.1 | 5 | 0-45 | 0-73 | 0-1867 | 0-3023 | Lipid transport |
| P02749 | Apolipoprotein H (beta-2-glycoprotein 1) | APOH_271 | ATVVYQGER | 0.02 | 2 | 3-19 | 3-30 | 124-857 | 122-1318 | Lipid transport, anticoagulation |
| P61769 | Beta-2-microglobulin | B2MG_69 | VEHSDLSFSK | 1 | 25 | 38-77 | 31-114 | 332-1220 | 493-1804 | Innate immunity |
| P00450 | Ceruloplasmin | CERU_70 | ALYLQYTDETFR | 0.1 | 1 | 6-11 | 5-21 | 557-1528 | 712-2981 | Antioxidant, copper & iron homeostasis |
| P36222 | Chitinase-3-like protein 1 (YKL-40) | CH3L1_290 | EAGTLAYYEICDFLR | 1 | 5 | 1.4-2.9 | 1-7 | 28-141 | 53-355 | microglial activation |
| P10909 | Clusterin (ApoJ) | CLUS_183 | ASSIIDELFQDR | 0.1 | 2 | 59-79 | 48-96 | 2444-4771 | 2924-5778 | Lipid transport, chaperone |
| P10645 | Chromogranin A | CMGA_322 | SGELEQEEER | 0.02 | 2 | 9-28 | 4-52 | 473-1655 | 248-3037 | Prohormone |
| Q12860 | Contactin 1 | CNTN1_172 | WLLNEFPVFITMDK | 5 | 5 | 6.5-8.3 | 3-24 | 53-137 | 286-3778 | Cell adhesion, signaling between axons and glia, NOTCH activation |
| Q02246 | Contactin 2 | CNTN2_168 | WLLNEFPNFIPTDGR | 0.2 | 2 | 0.4-1 | 0.4-2.5 | 986-3269 | 51-328 | Cell adhesion, axon guidance |
| P01024 | Complement component C3 | CO3_1172 | AGDFLEANYMNLQR | 0.1 | 0.2 | 8-15 | 9-37 | 656-4765 | 1884-8045 | Complement activation |
| P0C0L4 | Complement component C4 | CO4A_COB_1085 | VLSLAQEQVGGSPEK | 0.02 | 1 | 9-22 | 7-27 | 247-1424 | 1603-6017 | Propagation of complement pathway |
| P01034 | Cystatin C | CYTC_35 | VGGPMDASVEEEGVR | 0.1 | 0.2 | 21-78 | 17-120 | 67-163 | 315-2173 | Cysteine proteinase inhibitor |
| O95502 | Neuronal pentraxin receptor | NPTXR_22 | IIASVPLAASPAR | 0.004 | 1 | 1.2-2.7 | 0.8-4 | 392-1143 | 50-235 | Uptake of synaptic material |
| Q92823 | NrCAM | NRCAM_806 | YIVSGTPTFVPYLIK | 0.1 | 1 | 3-7 | 2-12 | 392-1143 | 341-1974 | Cell adhesion, neurite outgrowth (with contactin) |
| P00747 | Plasminogen | PLMN_681 | VIPACLPSPNYVVADR | 0.002 | 0.02 | 0.5-2.4 | 0.5-4 | 36-250 | 48-436 | Extracellular matrix maintenance |
| P04156 | Prion protein | PRIO_195 | GENFTETDVK | 0.1 | 5 | 0.5-1.4 | 0.4-1.8 | 8.3-44 | 13-58 | Myelination, copper homeostasis, cell signaling, cell adhesion, synaptogenesis |
| P04156 | Prion protein | PRIO_209 | VVEQMCITQYER | 0.1 | 1 | 0.7-2.3 | 0.4-3 | 13-73 | 13-94 | Myelination, copper homeostasis, cell signaling, cell adhesion, synaptogenesis |
| P41222 | Prostaglandin-d2 synthase | PTGDS_23 | APEAQVSVQPNFQQDK | 1 | 1 | 357-535 | 318-709 | 6377-12930 | 7702-14139 | Neuromodulator, vasodilator production |
| P02753 | Retinol binding protein | RET4_108 | YWGVASFLQK | 0.5 | 0.5 | 8-16 | 4-26 | 88-418 | 111-683 | Retinol carrier, binds transthyretin |
| P08294 | Superoxide dismutase | SODE_177 | AVVVHAGEDDLGR | 0.02 | 1 | 2-4 | 1.5-4.6 | 41-114 | 44-136 | Antioxidant |
| P05452 | Tetranectin | TETN_51 | LDTLAQEVALLK | 0.02 | 0.1 | 4-10 | 3-15 | 61-259 | 73-382 | Cell structure |
| P02787 | (sero)transferrin | TRFE_216 | DGAGDVAFVK | 0.5 | 5 | 85-166 | 42-272 | 4947-14668 | 3697-24140 | Iron transport |
| P02766 | Transthyretin | TTHY_56 | AADDTWEPFASGK | 0.5 | 1 | 218-373 | 167-426 | 3989-6808 | 3043-7789 | Thyroxin and retinol transport |
| P62760 | Visinin-like protein 1 | VISL1_101 | LNWAFNMYDLDGDGK | 0.2 | 0.2 | <LOD | <LOD | <LOD | <LOD | Calcium sensor, neuronal injury marker |
| P62760 | Visinin-like protein 1 | VISL1_119 | VEMLEIIEAIYK | 2 | 5 | <LOD | <LOD | <LOD | <LOD | Calcium sensor, neuronal injury marker |
| P62760 | Visinin-like protein 1 | VISL1_43 | LNLEEFQQLYVK | 1 | 1 | <LOD | <LOD | <LOD | <LOD | Calcium sensor, neuronal injury marker |
| P68082 | Equine myoglobin | Myo_horse_65 | HGTVVLTALGGILK | 0.5 | 5 | n/a | n/a | n/a | n/a | Control, Internal Standard |
| P04114 | Apolipoprotein B | APOB_779 | ILGEELGFASLHDLQLLGK | 2 | 10 | n/a | n/a | n/a | n/a | Control, blood contamination |
| P00915 | Carbonic anhydrase 1 | CAH1_82 | GGPFSDSYR | 0.2 | 5 | n/a | n/a | n/a | n/a | Control, blood contamination |
| P00918 | Carbonic anhydrase 2 | CAH2_133 | AVQQPDGLAVLGIFLK | 1 | 10 | n/a | n/a | n/a | n/a | Control, blood contamination |
| P68871 | Hemoglobin subunit beta | HBB_106 | LLGNVLVCVLAHHFGK | 1 | 10 | n/a | n/a | n/a | n/a | Control, blood contamination |

Limit of detection (LOD) is defined as the lowest standard concentration with a signal to noise ratio (S/N) above the average S/N of the blank plus 2 times the standard deviation. Limit of quantitation (LOQ) is defined as the lowest standard concentration within 80-120% accuracy and <20%CV. Values represent the median of 4 different calibration curves prepared on different days run in duplicate or triplicate. For all peptides listed in the table the intra -and inter-assay CVs were <20%.

**Supplemental Table 2.** CSF total protein and Aβ_42_ do not correlate with chromogranin (CMGA), neuronal pentraxin receptor (NPTXR) or NrCAM, but tau and p-tau_181_ do (Spearman rank correlations in AD patients at baseline).

|  | CMGA | NPTXR | NrCAM |
| --- | --- | --- | --- |
| Total protein (n=32) | -0.22 | -0.22 | -0.12 |
| Aβ_42_ (n=21) | 0.05 | -0.01 | -0.02 |
| Tau (n=21) | 0.69 | 0.71 | 0.74 |
| p-tau_181_ (n=21) | 0.55 | 0.57 | 0.59 |
