# Supplementary figures and images for: Identification of longitudinally dynamic biomarkers in Alzheimer’s disease cerebrospinal fluid by targeted proteomics
Source: Mol Neurodegener. 2014 Jun 6;9:22. doi: 10.1186/1750-1326-9-22 (PMC4061120; doi:10.1186/1750-1326-9-22)

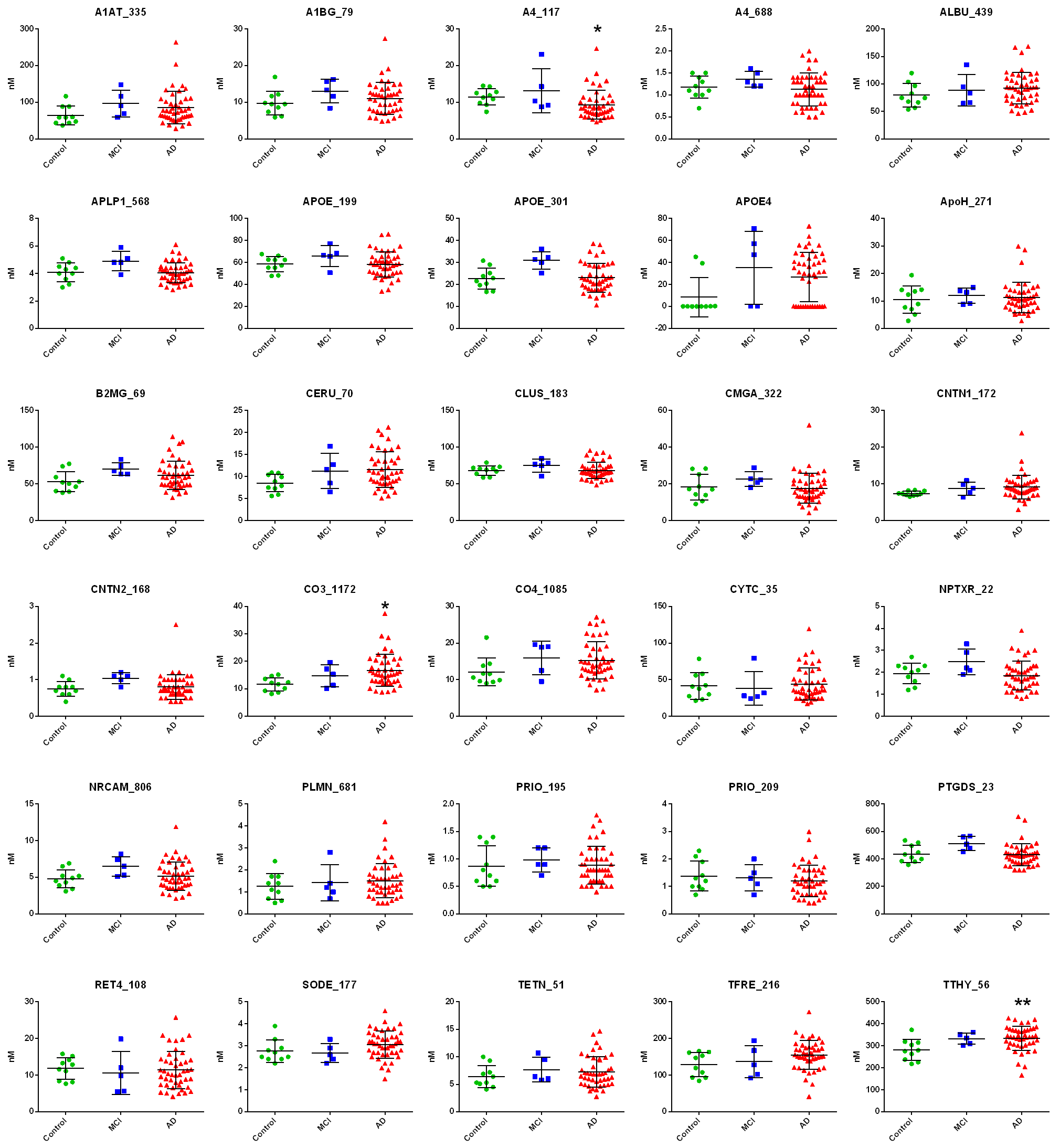

Supplement: Additional file 3: Figure S2 — Comparison of levels of detectable peptide biomarkers with inter-assay CVs of <20% in aged (>60y) cognitively-normal controls (n = 10), MCI (n = 5), and AD (n = 45) individuals. Differences between control and AD that reached significance are indicated with an asterisk (*p = 0.01-0.05, **p = 0.001-0.01, ***p < 0.001, linear regression comparison of log values corrected by the Benjamini & Hochberg method) (Control, green-circle, MCI blue-square, AD red-triangle). [file 1750-1326-9-22-S3.tiff]
